# Retraction Note: Establishment of an *Acanthamoeba* keratitis mouse model confirmed by amoebic DNA amplification

**DOI:** 10.1038/s41598-025-86535-5

**Published:** 2025-01-28

**Authors:** Heekyoung Kang, Hae‑Jin Sohn, A‑Young Park, A‑Jeong Ham, Jeong‑Heon Lee, Young‑Hwan Oh, Yong‑Joon Chwae, Kyongmin Kim, Sun Park, Hongseok Yang, Suk‑Yul Jung, Jong‑Hyun Kim, Ho‑Joon Shin

**Affiliations:** 1 Department of Microbiology, Ajou University School of Medicine, Suwon, 16499 Republic of Korea; 2 Department of Biomedical Science, Graduate School of Ajou University, Suwon, 16499 Republic of Korea; 3 Yonsei Eye Clinic, Yeongtong-gu Bongyeong-ro 1606, Suwon, 16704 Republic of Korea; 4 Department of Biomedical Laboratory Science, Molecular Diagnostics Research Institute, School of Health and Medicine, Namseoul University, Cheonan, 31020 Republic of Korea; 5 Institute of Animal Medicine, College of Veterinary Medicine, Gyeongsang National University, Jinju, 52828 Republic of Korea; 6 Present Address: Department of Tropical Medicine and Parasitology, Institute of Endemic Diseases, Seoul National University College of Medicine, Seoul, 03080 Republic of Korea

Retraction of: *Scientific Reports* 10.1038/s41598-021-83738-4, published online 18 February 2021

The Editors have retracted the Article.

After publication, concerns were brought to the attention of Editors about the published figures, and included apparent image duplication and rotation in Figure 5. The Authors provided alternative images but the dates on which the images were generated did not match the descriptions in the Article. The Editors have therefore lost confidence in the results and conclusions of this Article.

The Authors did not respond to correspondence from the Publisher about this retraction.

